# Scleral Fixation of Toric Intraocular Lens in the Absence of Capsular Support

**DOI:** 10.1155/2024/7157592

**Published:** 2024-04-03

**Authors:** Karolina Krix-Jachym, Natalia Błagun, Marek Rękas

**Affiliations:** Ophthalmology Department, Military Institute of Medicine-National Research Institute, Szaserów Street 128, 04-141 Warsaw, Poland

## Abstract

The study is aimed at describing a technique for scleral fixation of toric intraocular lens (TIOL) in the eyes without capsular support coexisting with corneal astigmatism. A monofocal toric hydrophobic lens with eyelets at the optic-haptic junction (enVista One-Piece Hydrophobic Acrylic MX60T Toric IOL; Bausch & Lomb) was fixated to the sclera using two fragments of 6–0 polypropylene monofilament, the ends of which were brought out through the sclera and cauterized. The astigmatic axis of a TIOL was adjusted according to the corneal astigmatic axis of the patient. The surgery was performed in the 5 eyes of 5 patients without capsular support. The method was safe and effective in fixing the lens to the sclera, and it ensured good centration of TIOL with predictable refractive outcomes. No conjunctival sutures, glue, or flap formation was required during the surgery. There were no relevant complications related to the procedure.

## 1. Introduction

Conventional scleral suture fixation of a toric intraocular lens (TIOL) is possible in the eyes without adequate capsular support with coexisting corneal astigmatism. However, the high risk of lens decentration and unpredictable refractive outcomes limit TIOL implantation in such cases.

In the eyes with a subluxated crystalline lens, in which the lens capsule can be preserved intraoperatively, a toric intraocular lens can be implanted in the capsular bag and the capsular bag-TIOL complex can be fixated to the sclera with iris retractors, using the technique previously described [1].

Several techniques of fixing a TIOL to the sclera in the absence of capsular bag have been reported [2], and various types of TIOLs were used, but a perfect solution to correct cylindrical refractive error in aphakic eyes has not been found.

The steep axis of the toric MX60T IOL is linear with the eyelets, which is crucial to align the lens with the axis of astigmatism [3]. Due to its design, the lens is suitable for “pseudo-4-point fixation” with scleral sutures [4].

In this study, we present a modified technique of scleral suture fixation of enVista TIOL and its introductory clinical outcomes. The demonstrated approach requires no scleral flaps, conjunctival sutures, or glue. This method was developed based on the principles of the Yamane technique [5] and offers TIOL stable support.

## 2. Surgical Technique

**Figure 1:**
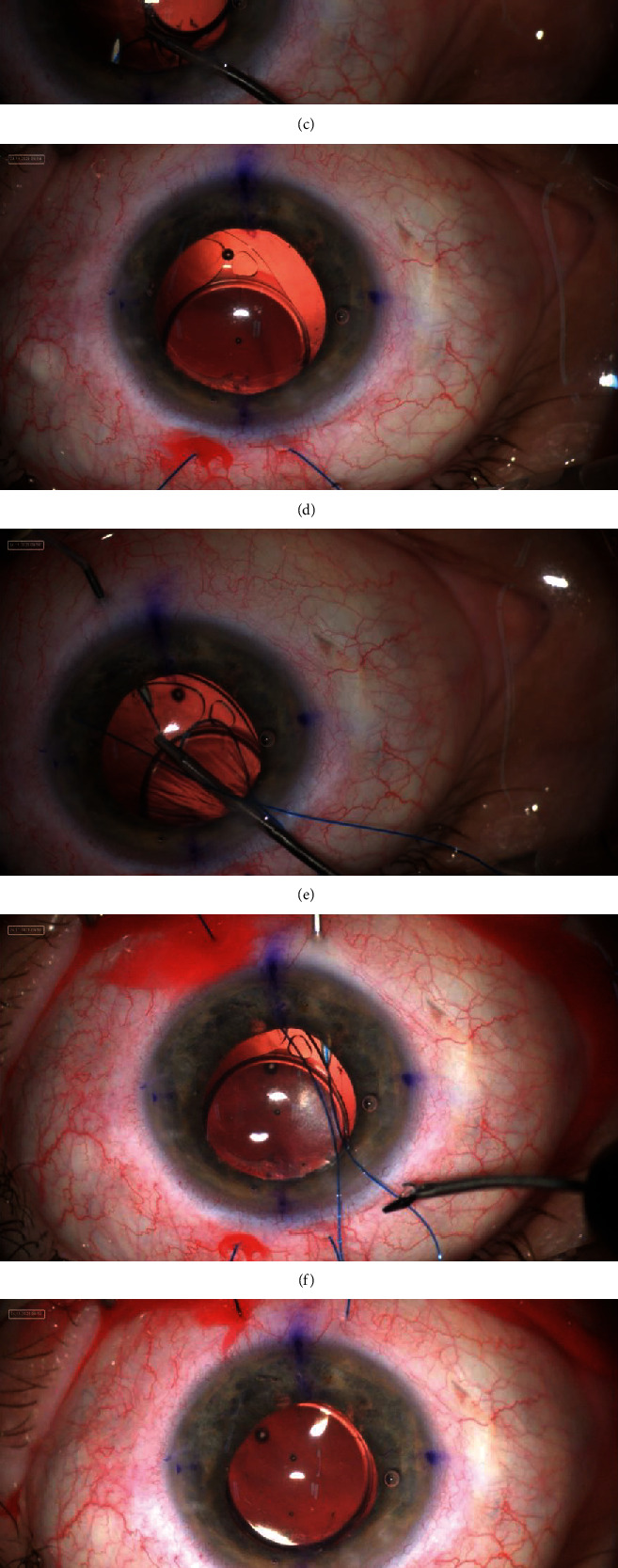
(a–i) Surgical technique of scleral fixation of toric intraocular lens in the absence of capsular support with the use of “pseudo-4-point” technique.

Each surgery was performed under retrobulbar anesthesia. The procedure began with marking the steep axis of astigmatism on the cornea. Four attachment points were symmetrically located on the sclera (two in superior and two in inferior quadrants), 2 mm posteriorly to the limbus, 6 mm apart, with the astigmatic axis in the center. Then, 2.2 mm temporal clear corneal incision and additional, superonasal port were created. Anterior vitrectomy was performed when needed. A one-piece foldable hydrophobic acrylic TIOL (Bausch & Lomb, Inc., Rochester, NY, USA) (enVista MX60T) was inserted to the anterior chamber after injection of viscoelastic material. The axis of the TIOL was set according to the steep axis of the astigmatism (Figure 1(a)). In addition, the axis of the lens was always located centrally between the planned needle drop points on the sclera. Then, a needleless fragment of 6–0 polypropylene monofilament suture (Ethicon Inc., Somerville, NJ) was placed in the anterior chamber through the main incision. Next, in the superonasal quadrant, a 27-gauge needle was inserted through the sclera to the anterior chamber. The needle was advanced posteriorly to the iris until its end was seen through the pupil. The end of the suture was carried out through the IOL eyelet and inserted into the lumen of the needle (Figure 1(b)) and then externalized. A second (superotemporal) sclerotomy using the same needle was performed about 6 mm from the first one, and the procedure was repeated with the second end of the suture (Figure 1(c)). After externalization of both ends of the suture (Figure 1(d)), the same steps were used to externalize the ends of the second suture in the inferotemporal and inferonasal quadrants of the operated eye (Figures 1(e)–1(g)). The position of the TIOL was additionally adjusted by pulling the upper ends of the sutures. The alignment between the conjunctival marking for the steep axis and that of the TIOL optic was checked. The ends of the diagonally opposite sutures were trimmed and cauterized with an ophthalmic cautery device to create flanges (Figure 1(h)). The procedure was then repeated with the remaining ends of the sutures. Four half-sphere-shaped melted tips of the sutures were then withdrawn and fixed subconjunctivally (Figure 1(i)).

## 3. Materials and Methods

The described technique was performed in the 5 eyes of 5 patients. The Institutional Review Board approved the study, and all patients provided informed consent; the tenets of the Declaration of Helsinki were followed. The indication for scleral fixation of TIOL was the lack of adequate capsular support with coexisting corneal astigmatism greater than or equal to 2 diopters. Aphakia and lack of capsular support had diverse etiology (Table 1). Exclusion criteria included irregular astigmatism, all cases of corneal opacity, and previous corneal surgery. This nonrandomized, prospective study was performed in 2021 in the Ophthalmology Department, Military Institute of Medicine-National Research Institute, Warsaw, Poland, and all operations were performed by one surgeon (MR). The TIOL implantation technique was identical in all cases. The postoperative treatment typically included antibiotic and anti-inflammatory topical drugs. The follow-up duration was 3.6 months (range 3-4 months). At follow-up, a thorough ophthalmic examination was performed and included uncorrected visual acuity (UCVA), corrected distance visual acuity (CDVA), preoperative corneal cylinder (IOLMaster 700; Carl Zeiss Meditec AG, Jena, Germany), postoperative refractive cylinder, slit lamp evaluation, applanation tonometry, Anterion exam, fundus evaluation, and B-scan ultrasonography (when the fundus was not visible). Data on the operated eye, surgical technique, and complications was also collected.

## 4. Results

The technique was performed in the 5 eyes of 5 patients with corneal astigmatism of more than 2 D (mean 2.66 diopters; ±0.49 (SD) (range 2.00-3.25 D)). The mean age of patients was 55 ± 14.01 SD (range 37-79 years). The mean follow-up was 3.6 months (±0.49 (SD) (range 3-4 months)). Demographic and clinical data are summarized in Table 1.

The mean surgical time was 40 minutes (range 25-50 min). The clinical investigations showed good results (Table 2). The postoperative period was uncomplicated in regard to visual function. The mean preoperative and postoperative corrected distance visual acuities (CDVA) on the Snellen chart were 0.4 ± 0.2 (SD) (range 0.1-0.8) and 0.9 ± 0.2 (SD) (range 0.5-1.0), respectively. The mean preoperative and postoperative IOPs were 17.4 mmHg ± 4.63 (SD) (range 13–24 mmHg) and 17 mmHg ± 2.45 (SD) (range 14-21 mmHg), respectively. The mean preoperative corneal astigmatism was 2.66 diopters (±0.49 (SD) (range 2.00-3.25 D)), while mean postoperative refractive astigmatism was 0.9 diopters (±0.12 (SD) (range 0.75-1.0 D)), respectively. A minimal corneal edema was present in the 2 eyes (40%) (cases 2 and 4) and resolved after 1 week. The mean endothelial cell count was 2236.6 cells/mm^2^ before the surgery and 2128 cells/mm^2^ on the last visit. The endothelial cell loss was 4.9% during the follow-up. None of the cases showed cystoid macular edema. The one eye (20%) developed raised intraocular pressure that responded well to medical therapy (case 2), and the one eye showed no improvement in CDVA because of concomitant pathology (amblyopia accompanying choroidal coloboma). Mild vitreous haemorrhage occurred in the 2 eyes (40%) (cases 1 and 5) and resolved spontaneously shortly after surgery. The TIOL position remained stable in all cases within the observation period. An Anterion examination during a recent visit revealed well-positioned TIOL in all eyes. No other postoperative complications were encountered.

### 4.1. Case 1

**Figure 2:**
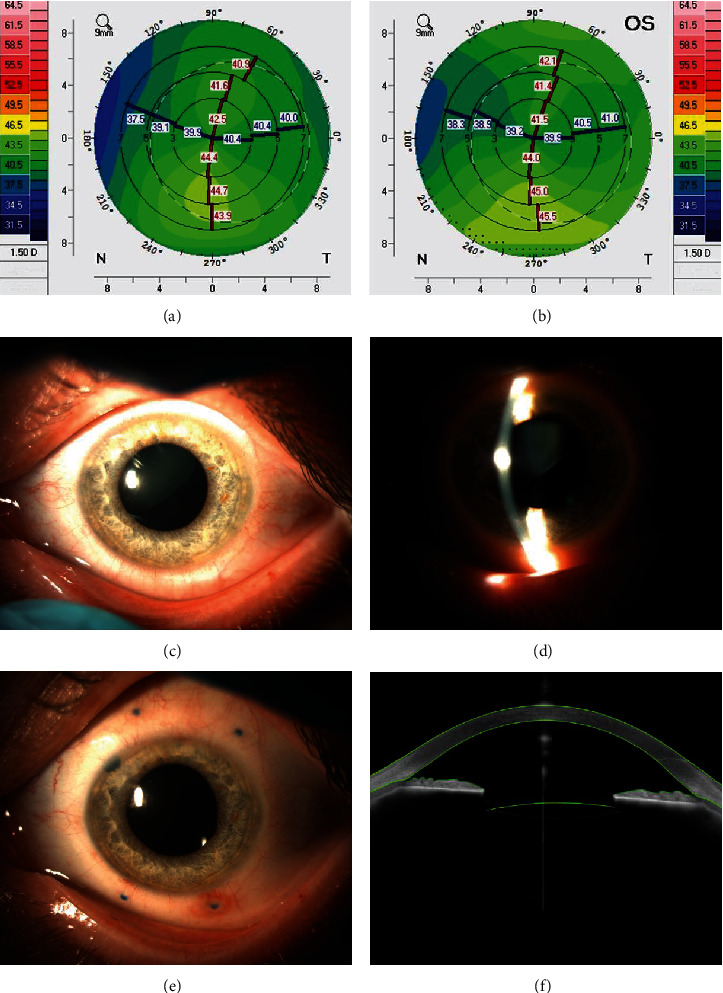
Case 1: (a, b) the Scheimpflug tomography shows corneal astigmatism; (c, d) the left eye before surgery–lens subluxation; (e) slit lamp photography 1 month after surgery; (f) postoperative Anterion examination shows IOL position after surgery.

A 37-year-old man with bilateral lens subluxation presented with a deterioration of vision over 5 years with astigmatism and myopia in both eyes. In his left eye, CDVA was initially 20/4000, and the corneal astigmatism was 3.0 diopters. TIOL in-the-bag implantation was planned but abandoned due to capsule rupture. Therefore, the TIOL was supported by a “pseudo-four-point” scleral fixation as described above (Figure 2). After 4 months, CDVA was 20/20, and the residual astigmatism was 1.00 Dcyl.

### 4.2. Case 2

A 44-year-old woman with aphakia in her left eye underwent phacoemulsification and TIOL implantation 6 years ago, PPV for retinal detachment 5 years ago, and subsequently PPV for PC-TIOL luxation to the vitreous cavity 6 months ago. Before surgery, the CDVA was 20/40, and the corneal astigmatism was 2.9 D. Four months after surgery, CDVA was 20/25 and the IOP was 18 mmHg on two antiglaucoma topical drugs started because of raised IOP during follow-up. The residual astigmatism was 1.0 Dcyl.

### 4.3. Case 3

A 66-year-old man was examined due to IOL dislocation in his right eye. There was phacoemulsification and ocular diseases (PEX and secondary glaucoma treated with sclerectomy in both eyes about 5 years ago) in anamnesis. In the first step, subluxated IOL was explanted. CDVA prior to TIOL scleral fixation was 20/25. The preoperative corneal astigmatism was 2.00 D. Three months after surgery, UCVA was 20/20, and the refractive error was −0.50–0.75 × 177.

### 4.4. Case 4

A 50-year-old man was referred from another clinic with decreased VA and subluxation of multifocal TIOL in his left eye. Phacoemulsification with multifocal TIOL implantation was performed 4 months earlier. Reposition of IOL was performed three times, and a suture fixation of the multifocal TIOL was also attempted in a primary clinical center. Initial CDVA was 20/70, and the corneal astigmatism was 2.15 D. The patient was qualified for multifocal TIOL explantation and simultaneous TIOL scleral fixation. Three months after surgery, UCVA was 20/20 and the refractive error was +0.50–0.75 × 51.

### 4.5. Case 5

**Figure 3:**
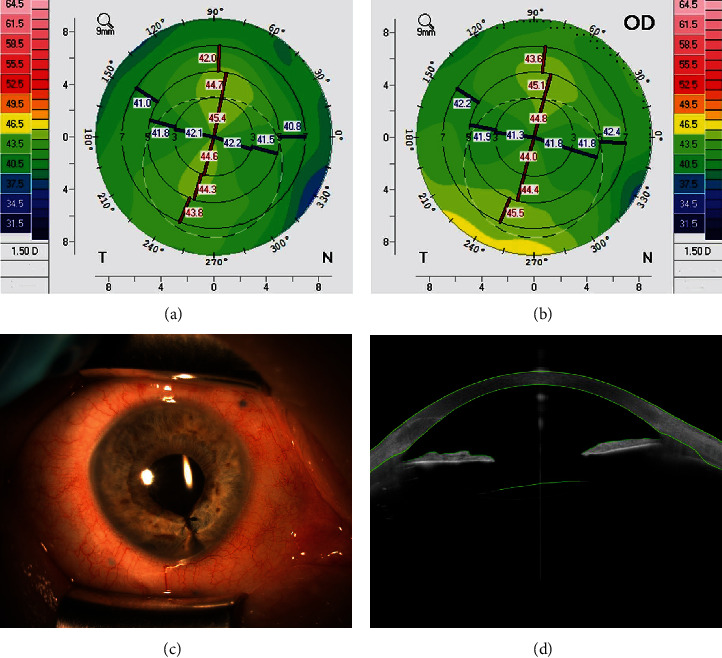
Case 5: (a, b) the Scheimpflug tomography shows corneal astigmatism; (c) slit lamp photography 4 months after surgery; (d) postoperative Anterion examination shows IOL position after surgery.

A 79-year-old woman presented with aphakia in her right eye. She underwent phacoemulsification surgery and closure of iris coloboma 1 month earlier in our department. CDVA was 20/40. Preoperative corneal astigmatism was 3.25 diopter. Scleral fixation of TIOL was performed. Four months after surgery, UCVA was 20/40 due to amblyopia coexisting with choroidal coloboma. The postoperative refractive error was +1.50–1.00 × 95. There was no significant tilt or decentration of TIOL (Figure 3).

## 5. Discussion

TIOLs require precise centration and good stability to decrease refractive astigmatism postoperatively. Difficulty in achieving these limits the common use of scleral fixation of TIOLs in the eyes without capsular support, despite the fact that various techniques have been previously reported [2, 6–9].

In 2009, Borkenstein et al. showed that a transscleral fixation of a TIOL (Rayner 570T) in an aphakic eye is possible and can result in improved visual rehabilitation [2]. Later, Emanuel et al. described repositioning and suture fixation of a TIOL (SN6AT series; Alcon Laboratories, Inc., Fort Worth, TX) [6]. Another proposition was that of Kelkar et al., who described a sutureless, glueless scleral fixation of a single-piece TIOL (Tecnis) [8]. Karadag et al., on the other hand, fixed a plate haptic one-piece TIOL (Acriva BB T UDM 611, VSY Biotechnology, Amsterdam, Netherlands) to the sclera using 10–0 double-armed propylene suture [9].

In 2015, Yang et al. described the results of scleral fixation of a nontoric model of the enVista MX60, Bausch & Lomb, Inc. IOL, using the eyelets located at the optic-haptic junction for suture placement [10].

In 2021, Ward et al. described a scleral fixation technique of a sutured eyelet TIOL. The authors used two-point suture scleral fixation of MX60T TIOL. Prior to insertion, the haptics were trimmed distal to the eyelet [3].

Due to the fact that two-point fixation makes postoperative outcomes unpredictable and increases the risk of IOL tilt [11–14], the four-flanged technique for fixating TIOLs to the sclera appears worthy of notice. Canabrava et al. introduced a four-flanged intrascleral IOL fixation technique with 5–0 polypropylene that does not require flap creation, suture knots, or glue [15]. Four fixation points were created by melting the tips of the suture, thereby creating flanges over the sclera. The authors used a nontoric model of IOL.

In this study, MX60T TIOL was used for the management of astigmatism in eyes without capsular support. MX60T TIOL allows a “pseudo-4-point” fixation and can be delivered through an incision size of 2.2 mm to limit potential SIA (surgically induced astigmatism). The suture was looped over the haptic, under the eyelet, and then back over the haptic, as it was previously described for the nontoric model, ensuring that the IOL remains stable with no tilt after externalization of the sutures [16].

The use of MX60T model of TIOL together with the technique described above in the eyes that require scleral fixation offers numerous advantages. Intraoperatively, the lens can be centered in the eye easily by adjusting the tension on the sutures. Cauterization of the suture ends produces the flanges which hold the IOL in place. “Pseudo-four-point” fixation gives adequate support for the lens without the risk of axis change after surgery. The procedure is relatively simple and short as there is no need to create flaps or knots.

Every patient in this study has demonstrated the right TIOL centration postoperatively with a reduction in astigmatism. There were no surgical complications that resulted in deterioration of visual outcomes clinically. However, it is necessary to conduct more studies with larger groups of patients and longer follow-up to evaluate the long-term safety and stability of the procedure. More prevalent use of premium IOLs in the eyes without capsular support after previous interventions remains a goal to be achieved in the meantime.

## Figures and Tables

**Table 1:** Demographic and clinical data of patients with aphakia and corneal astigmatism managed with TIOL scleral fixation.

Case	Sex	Eye	Age	Etiology	Follow-up	Complication
1	M	OS	37 years	Subluxation of the lens	4 months	Mild vitreous haemorrhage
2	F	OS	44 years	IOL luxation to the vitreous cavity	4 months	Corneal edema, high IOP
3	M	OD	66 years	Subluxation of the IOL	3 months	None
4	M	OS	49 years	Subluxation of the IOL	3 months	Corneal edema
5	F	OD	79 years	Aphakia	4 months	Mild vitreous haemorrhage

F: female; M: male; OD: right eye; OS: left eye; IOL: intraocular lens; IOP: intraocular pressure.

**Table 2:** Follow-up data of patients after scleral fixation of TIOL.

Case	Preoperative									Postoperative		
	Diagnosis	UDVA	CDVA	MR	Axial length (mm)	Flat corneal power (diopter)	Steep corneal power (diopter)	enVista TIOL type	TIOL power (diopter)	UDVA	CDVA	MR
1	Lens subluxation	20/400	20/200	−8.75 Ds × −6.0 Dc axis 165	23.27	40.37	43.42	MX60TP 425+280	+28.0	20/30	20/20	−3.00 Ds × −1.00 Dc axis 48
2	Aphakia	20/4000	20/40	+7.00 Ds × −2.00 Dc axis 170	26.35	43.32	46.23	MX60TP 425+115	+11.5	20/30	20/25	−0.00 Ds × −1.0 Dc axis 83
3	IOL dislocation	20/4000	20/25	+12.50 Ds × −1.00 Dc axis 24	23.46	42.31	43.89	MX60TP 275+225	+22.5	20/25	20/20	−0.50 Ds × −0.75 Dc axis 177
4	IOL dislocation	20/200	20/70	+1.00 Ds × −3.25 Dc axis 20	24.31	40.80	42.95	MX60TP 275+210	+21.0	20/20	20/20	+0.50 Ds × −0.75 Dc axis 51
5	Aphakia	20/4000	20/40	+15.25 Ds × −2.00 Dc axis 160	22.13	42.11	45.71	MX60TP 500+260	+26.0	20/70	20/40	+1.50 Ds × −1.00 Dc axis 95

UDVA: uncorrected distance visual acuity; CDVA: corrected distance visual acuity; MR: manifest refraction; TIOL: toric intraocular lens; IOL: intraocular lens; Ds: diopter sphere; Dc: diopter cylinder.

## Data Availability

Data is available on request (from corresponding author).

## References

[1] Krix-Jachym K. M., Błagun N., Kicińska A. K., Dyda W., Rękas M. T. (2022). Sutureless technique for repositioning and scleral fixation of the capsular bag–intraocular lens complex with permanent use of iris retractors. *Journal of Cataract & Refractive Surgery*.

[2] Borkenstein A. F. M., Reuland A., Limberger I.-J., Rabsilber T. M., Auffarth G. U. (2009). Transscleral fixation of a toric intraocular lens to correct aphakic keratoplasty with high astigmatism. *Journal of Cataract and Refractive Surgery*.

[3] Ward M. S., Hou A. C., Murphy D. A., Schmutz M. A., Riaz K. M. (2021). Scleral fixation of a toric lens to treat corneal astigmatism in eyes without capsular support. *Clinical Ophthalmology*.

[4] Watane A., Botsford B. W., Sood A. B. (2021). Scleral-sutured intraocular lens dislocations secondary to eyelet fractures. *American Journal of Ophthalmology*.

[5] Yamane S., Sato S., Maruyama-Inoue M., Kadonosono K. (2017). Flanged intrascleral intraocular lens fixation with double-needle technique. *Ophthalmology*.

[6] Emanuel M. E., Randleman J. B., Masket S. (2013). Scleral fixation of a one-piece toric intraocular lens. *Journal of Refractive Surgery*.

[7] Jun J. H., Bang S. P. (2018). Transscleral fixation of a toric intraocular lens by a slipable suture technique. *Canadian Journal of Ophthalmology*.

[8] Kelkar A., Shah R., Kelkar J., Kelkar S., Arora E. (2015). Sutureless, glueless, scleral fixation of single-piece and toric intraocular lens: a novel technique. *Case Reports in Ophthalmology*.

[9] Karadag R., Gunes B., Aykut V., Oguz H., Demirok A. (2018). Scleral suture fixation technique for dislocated plate haptic toric IOL. *International Ophthalmology*.

[10] Yang J. M., Yoon K. C., Ji Y. S. (2015). Transscleral fixation of single-piece foldable acrylic lens with eyelets at the optic-haptic junction. *Canadian Journal of Ophthalmology*.

[11] Whang W. J., Kwon H., Jeon S. (2020). Application of a four-flanged intrascleral fixation technique for toric and multifocal intraocular lenses. *American Journal of Ophthalmology Case Reports*.

[12] Das S., Nicholson M., Deshpande K., Kummelil M. K., Nagappa S., Shetty B. K. (2016). Scleral fixation of a foldable intraocular lens with polytetrafluoroethylene sutures through a Hoffman pocket. *Journal of Cataract and Refractive Surgery*.

[13] Liu H. T., Jiang Z. X., Tao L. M. (2016). New two-point scleral-fixation technique for foldable intraocular lenses with four hollow haptics. *International Journal of Ophthalmology*.

[14] Steinert R. F., Brint S. F., White S. M., Fine I. H. (1991). Astigmatism after small incision cataract surgery. *Ophthalmology*.

[15] Canabrava S., Andrade N., Rezende P. H. (2021). Scleral fixation of a 4-eyelet foldable intraocular lens in patients with aphakia using a 4-flanged technique. *Journal of Cataract and Refractive Surgery*.

[16] Aderman C., Regillo C. D. (2018). *Succeeding with scleral-sutured lenses*.

